# POEM-based endoscopic myotomy for esophageal diverticulum: a case series study

**DOI:** 10.1186/s12876-026-04910-6

**Published:** 2026-05-12

**Authors:** Kehan Li, Cheng Guo, Bensong Duan, Tao Chen, Meidong Xu

**Affiliations:** Endoscopy Center, Department of Gastroenterology, Shanghai East Hospital, Tongji University School of Medicine, No.150 Jimo Road, Pudong New District, Shanghai, 200120 People’s Republic of China

**Keywords:** Esophagus diverticulum, Gastroscopy, Peroral endoscopic myotomy, Case series

## Abstract

**Background and aims:**

In recent years, the submucosal tunneling principle underlying Peroral endoscopic myotomy (POEM) has emerged as a minimally invasive endoscopic option for the treatment of esophageal diverticulum (ED). However, evidence regarding its effectiveness across different types of ED remains limited. This study aimed to evaluate the clinical efficacy and safety of endoscopic myotomy for the management of esophageal diverticulum.

**Methods:**

This single-center retrospective case series included patients with ED who underwent POEM-based endoscopic myotomy at the East Hospital Affiliated to Tongji University between February 2019 and February 2023.

**Results:**

Seven patients (aged 44 to 80 years; four males) were included, comprising three with epiphrenic diverticulum, three with Zenker diverticulum (ZD), and one with mid-esophageal diverticulum. Different subtypes received tailored POEM-based procedures: epiphrenic diverticula underwent D-POEM; two cases with concomitant achalasia received combined POEM and D-POEM; ZD patients underwent z-POEM; and the mid-ED case received tunnel-technique myotomy. All procedures were technically successful, with mean operative times of 84 min (epiphrenic), 23 min (ZD), and 30 min (mid-ED). Intraoperative assessment demonstrated complete flattening of the diverticulum without residual fluid, and no clinical recurrence was documented during a median follow-up of 280 days.

**Conclusions:**

POEM-based endoscopic myotomy appears to be a feasible and minimally invasive treatment option for selected patients with ED. In patients with concomitant achalasia, combined POEM and diverticular myotomy may be feasible in selected cases.

**Supplementary Information:**

The online version contains supplementary material available at 10.1186/s12876-026-04910-6.

## Introduction

Esophageal diverticulum (ED) is a rare structural abnormality of the esophagus that can be categorized into three major subtypes based on anatomical location and underlying pathophysiology: Zenker diverticulum (ZD), mid-esophageal diverticulum (mid-ED), and epiphrenic diverticulum [1–3]. ZD is a pulsion diverticulum arising from the weak Killian’s triangle and is estimated to occur in approximately 0.01%–0.11% of the population, predominantly in elderly individuals [4]. Mid-ED is less common, accounting for 15% of all esophageal diverticula, most of which are traction-type lesions often associated with inflammatory or motility disorders [5]. Epiphrenic diverticulum is the rarest subtype, with a reported incidence of < 0.02%, and is strongly associated with esophageal motility disturbances such as achalasia or diffuse esophageal spasm [6]. While many cases are asymptomatic, symptomatic ED may lead to dysphagia, regurgitation, aspiration, chest pain, or weight loss, necessitating intervention [7, 8].

Traditional management relies on surgical treatment. Thoracoscopic or open surgical diverticulectomy combined with myotomy remains the standard approach for symptomatic ED [9, 10]. However, surgical management is associated with subintimal morbidity, including reported complication rates of 10–30% such as leakage, recurrent laryngeal nerve injury, and mediastinitis, as well as recurrence rates of up to 20–30% in non-Zenker diverticula. Previous surgical and endoscopic series have typically included 10–50 patients, highlighting the limited but evolving evidence base in this field [11, 12]. Despite advances in surgical techniques, large diverticula, elderly patients, and individuals with significant comorbidities continue to pose therapeutic challenges, highlighting the need for safer and less invasive alternatives [13].

With the evolution of therapeutic endoscopy, the submucosal tunneling technique has emerged as a minimally invasive strategy for ED. Several studies have demonstrated that Peroral endoscopic myotomy (POEM) and its derivatives—including D-POEM for epiphrenic diverticulum, Z-POEM for Zenker diverticulum, and tunnel-technique myotomy for mid-ED—have shown encouraging short-term outcomes with the advantages of minimally invasive access and faster recovery in selected reports [1, 14, 15]. Nevertheless, existing reports remain limited by small sample sizes, heterogeneous techniques, and insufficient comparative data across different ED subtypes, leaving uncertainty regarding the optimal procedural approach for each anatomical variant [16].

Given these gaps, the present study was designed as a retrospective case series to evaluate the feasibility, technical characteristics, and clinical outcomes of POEM-based endoscopic myotomy across three distinct types of ED. This methodological approach allows detailed characterization of procedural differences, operative complexity, and symptom resolution among D-POEM, Z-POEM, and mid-ED tunneling, thereby providing preliminary real-world data on procedural feasibility and short-term outcomes across different ED subtypes.

## Materials and methods

### Study design and patients

Patients were included if they had a confirmed diagnosis of Zenker diverticulum, mid-esophageal diverticulum, or epiphrenic diverticulum on endoscopy or barium esophagography and were considered suitable candidates for endoscopic intervention based on symptoms and anatomical characteristics. Patients with uncontrolled infection, significant cardiopulmonary contraindications to general anesthesia, or altered anatomy precluding safe creation of a submucosal tunnel were excluded. During the study period, esophageal diverticulum was infrequently encountered at our center. All symptomatic patients considered suitable for POEM-based endoscopic myotomy were consecutively enrolled. The small sample size therefore reflects the rarity of the disease and strict procedural selection. The mucosal entry was created proximal to the diverticular neck for tunnel access. The submucosal tunnel was then directed toward and along the diverticular septum or muscular ridge, with dissection centered on the distal/free margin of the septum to permit complete myotomy under direct visualization without intentional entry into the diverticular sac. Myotomy length was individualized according to diverticulum type; in general, septal myotomy was performed along the full length of the diverticular septum, and in patients with concomitant achalasia the myotomy was extended approximately 2–3 cm beyond the lower esophageal sphincter.

### Diagnostic evaluation

The diagnosis of ED in all patients was established through upper endoscopy and X-ray barium examination. Endoscopy allowed direct visualization of the diverticular lumen, while radiographic imaging confirmed the presence of a pouch-like outpocketing that communicated with the esophageal lumen, consistent with standard diagnostic definitions. When clinically relevant, esophageal motility disorders including achalasia were assessed using endoscopic features and radiographic morphology. High-resolution manometry was not routinely performed, and the diagnosis of achalasia in this cohort was based on endoscopic and radiographic findings rather than formal Chicago Classification-based high-resolution manometry criteria.

### Operative technique

All procedures were performed under general anesthesia with endotracheal intubation. A therapeutic endoscope fitted with a transparent distal cap was introduced into the esophagus, and a submucosal injection was administered proximally to the diverticular neck to create adequate mucosal elevation. A mucosal incision was then made to gain access to the submucosal layer. The tunnel was developed toward the diverticular ridge or septum and then centered along the distal/free margin of the septum, allowing exposure of the septal muscle while preserving the mucosa of the diverticular sac. In cases with concomitant achalasia, a single continuous submucosal tunnel was created, allowing septal myotomy along the full length of the diverticular septum and extension of the myotomy 2–3 cm beyond the lower esophageal sphincter.

Within this septum-oriented tunnel, the muscular architecture of the diverticulum became clearly exposed. Myotomy was carried out under direct visualization along the exposed septal or muscular ridge according to the diverticulum type: the septum between the diverticulum and the true esophageal lumen was divided in Zenker diverticulum (Z-POEM); the muscular ridge of epiphrenic diverticulum was incised using the D-POEM approach, with an extended myotomy across the lower esophageal sphincter when concomitant achalasia was present; and for mid-esophageal diverticulum, the tunnel technique allowed controlled exposure and incision of the muscular ridge. Hemostasis was maintained throughout the procedure using coagulation forceps, and the mucosal entry site was closed with endoscopic clips once myotomy was completed.

### Data collection

Clinical and procedural information was retrospectively obtained from electronic medical records. Preoperative variables included age, sex, symptom characteristics, diverticular morphology, diverticular size, diagnostic subtype, and the presence of esophageal motility disorders. Symptom assessment was based on patient-reported outcomes documented during routine follow-up visits and assessed by the treating physicians, rather than by a validated symptom scoring system. Clinical success was defined as resolution of presenting symptoms, complemented by endoscopic improvement, including diverticular flattening and absence of food retention at follow-up endoscopy. Recurrence was defined as recurrent dysphagia, regurgitation, or choking symptoms, with or without corresponding endoscopic evidence of persistent diverticular retention or food stasis. Routine postoperative barium esophagography was not systematically performed; therefore, radiologic improvement and radiologic recurrence were not used as formal study endpoints. Follow-up evaluation was conducted during outpatient visits, with endoscopic reassessment typically performed approximately 3–6 months post-procedure.

### Statistical analysis

Statistical analysis was conducted using descriptive analytical methods. Continuous variables were examined for distributional patterns and summarized as means with standard deviations when approximately normally distributed, or as medians with ranges when non-normally distributed. Categorical variables were reported as absolute counts and corresponding percentages. Given the small sample size, heterogeneity of diverticulum subtypes, and the observational nature of this case series, no comparative or inferential statistical tests were performed. All analyses were conducted to provide a descriptive characterization of clinical features, procedural details, and outcomes.

## Results

Seven patients were enrolled in this case series, including four females and three males, with a median age of 66 years (range, 44–80 years). The cohort consisted of three epiphrenic diverticula, three Zenker diverticula (ZD), and one mid-esophageal diverticulum (mid-ED). Dysphagia was the most common presenting symptom observed in five patients, while two patients reported reflux, choking cough, or a sensation of esophageal obstruction. Significant food retention was observed on baseline endoscopy in all patients. Diverticular characteristics varied by subtype: epiphrenic and mid-ED cases exhibited larger outpouchings (up to 3.3 × 2.5 cm), whereas ZD cases showed smaller yet deeper sac-like pouches, typically located 15–17 cm from the incisors (Table 1).

**Table 1:** Characteristics of the patients

	Sex	Age	Symptoms	Diverticular characteristics	Diagnosis
1	Female	44	Dysphagia	The cardia was about 36 cm away from the incisors. , and a huge diverticulum was seen near the cardia. The opening was 1.2 cm, and the diverticulum was about 3 × 3 cm. The cardia was narrow, and the endoscope could barely pass through.	Achalasia epiphrenic diverticulum
2	Male	58	Dysphagia	The middle and lower part of the esophagus was slightly dilated, and fluid retention was seen. The cardia was 40 cm away from the incisors. The cardia was narrow, and the endoscope could barely pass through. A huge diverticulum was seen near the cardia in the lower part.	Achalasia, epiphrenic diverticulum
*3*	Male	66	Dysphagia	A huge diverticulum was found at 31–34 cm from the incisors.	Epiphrenic diverticulum
*4*	Female	57	Reflux, choking cough, and esophageal obstruction feeling	A bag-like deep diverticulum was seen at the entrance of the esophagus 17 cm from the incisors, and food retention was observed.	ZD
*5*	Male	75	Reflux, choking cough, and esophageal obstruction feeling	A bag-like deep diverticulum was seen at the entrance of the esophagus 16 cm away from the incisor, and food retention was observed	ZD
*6*	Female	78	Reflux, choking cough, and esophageal obstruction feeling	A diverticulum was seen about 15 cm from the incisors, and Food retention was observed.	ZD
*7*	Female	80	Dysphagia	A pocket-like deep diverticulum was found at the entrance of the esophagus *25* cm from the incisor, and Food retention was observed.	*Mid ED*

Treatment selection was guided by diverticulum anatomy. All epiphrenic diverticula underwent D-POEM, and in two of these cases (1 and 2), coexisting achalasia necessitated the performance of a combined POEM and D-POEM procedure during the same operation. All three ZD cases were treated with z-POEM, while the single mid-ED case underwent tunnel-technique endoscopic myotomy. In this small cohort, achalasia was observed only in epiphrenic cases, which was consistent with the known association between distal diverticula and esophageal motility disorders (Table 2).

**Table 2:** General information of the patients D-POEM: diverticular peroral endoscopic myotomy; z-PEOM: Zenker’s peroral endoscopic myotomy

Case	Diverticular size (cm)	Motility disorder	Diverticular treatment	Operation duration (min)	Complication
1	3.1 × 2.2	Achalasia of cardia	D-POEM POEM	127	None
2	3.3 × 2.5	Achalasia of cardia	D- POEM POEM	90	None
*3*	2.5 × 3.0	None	D-POEM	30	None
*4*	2.0 × 2.3	None	z-PEOM	25	None
*5*	1.8 × 2.0	None	z-PEOM	15	None
*6*	2.3 × 2.5	None	z-PEOM	29	None
*7*	3.0 × 3.3	None	Tunnel technique myotomy of mid-esophageal diverticulum	30	None

All procedures were technically successful. Operative duration reflected anatomical complexity, averaging 84 min for epiphrenic diverticula, 23 min for ZD, and 30 min for mid-ED. No intraoperative or postoperative complications were reported across the cohort. Intraoperative assessment confirmed complete flattening of each diverticulum with free luminal flow, indicating successful septal or muscular correction. After a median follow-up of 280 days (range, 150–330 days), no clinical recurrence was documented during follow-up, and follow-up endoscopy showed resolution of food retention in all cases.

Detailed descriptions of the three representative cases and the corresponding figures are provided in the Supplementary Material.

## Discussion

In this retrospective case series, POEM-based endoscopic myotomy was technically successful in all seven patients with different subtypes of esophageal diverticulum. Short-term follow-up showed symptom improvement, resolution of food retention on follow-up endoscopy, and no documented clinical recurrence during a median follow-up of 280 days. These findings suggest that submucosal tunneling endoscopic myotomy may be a feasible minimally invasive option for selected patients with symptomatic ED [1]. Previously, ED resection was considered the standard treatment for conditions such as epiphrenic diverticulum, ZD, and symptomatic mid-ED [11, 17]. Still, performing surgery on the esophagus, particularly for epiphrenic diverticulum, is challenging because of its location in the mediastinum [18]. Moreover, traditional diverticular treatment, whether by endoscopic septotomy or external surgical approaches, may be associated with greater procedural trauma and procedure-related complications.

As endoscopic technology advances, submucosal tunneling techniques and POEM have been increasingly applied in the management of ED. In this approach, after proximal mucosal entry, the submucosal tunnel is directed toward the diverticular septum or muscular ridge and centered on the distal/free margin before myotomy is performed under direct endoscopic visualization. The D-POEM technique involves division of the septum between the diverticulum and the true esophageal lumen within a submucosal tunnel [18]. The mucosal entry is then closed with endoscopic clips rather than complete diverticular resection, as in open surgery. During the surgery, the diverticular septum or muscular ridge is incised to restore luminal passage and reduce food retention. Compared with conventional surgical approaches, the main advantage of D-POEM is its minimally invasive nature; however, direct comparative conclusions cannot be drawn from the present case series [19].

The present series adds to the growing clinical experience with endoscopic myotomy for ED. In our cohort, all procedures were completed successfully and no procedure-related adverse events were documented, which is consistent with previously reported endoscopic series. Although our findings support the technical feasibility and short-term clinical effectiveness of endoscopic tunnel myotomy, they should be interpreted cautiously because of the retrospective design, small sample size, and limited follow-up duration [20]. Previous studies have suggested that endoscopic myotomy may be associated with less procedural trauma, shorter recovery, and favorable short-term outcomes in selected patients with ED [21]. In the present study, follow-up endoscopy showed diverticular flattening without food retention in all treated patients. However, given the small number of cases and the relatively short follow-up period, our results should be regarded as preliminary, and longer-term follow-up is still required [15]. In patients with ED and achalasia, LES myotomy and diverticular septum incision can be performed within the same submucosal tunnel [22]. In case #1, submucosal lesions were identified during tunneling and were subsequently confirmed as leiomyomas by pathology and immunohistochemistry after resection. This finding was incidental and should be interpreted as a descriptive observation rather than a general procedural advantage. In our cohort, symptom improvement was observed after treatment, and no documented clinical recurrence was noted during the available follow-up period. This study has some limitations. First, only a small number of patients were included, which limits the generalizability of the findings [23]. Second, this was a retrospective single-center case series including only patients who underwent POEM-based tunneling treatment, which may have introduced selection bias. Third, the study lacked a control group, making it difficult to compare outcomes directly with other treatment approaches [24]. Fourth, potential confounding factors, such as medication history or comorbidities, were not systematically analyzed. Fifth, high-resolution manometry, standardized symptom scores, and routine postoperative barium esophagography were not systematically available, which limits the assessment of associated motility disorders, symptom severity, and objective radiologic outcomes. Finally, the follow-up duration was relatively short, which may have reduced the detection of late recurrence.

## Conclusion

In this case series, we demonstrated that submucosal tunneling endoscopic techniques—including D-POEM, z-POEM, and tunnel-technique myotomy—represent a safe, minimally invasive, and anatomically tailored treatment approach for different subtypes of esophageal diverticula. All seven patients achieved symptom improvement, diverticular flattening, and no documented clinical recurrence during a median follow-up of 280 days, with no intra- or postoperative complications. The coexistence of achalasia in epiphrenic diverticula in our cohort was consistent with the known association between distal diverticula and motility disorders, supporting the feasibility of combined POEM and D-POEM in selected cases. These findings support the clinical feasibility and potential therapeutic value of endoscopic tunnel myotomy in selected patients with symptomatic or anatomically significant diverticula. Despite the limitations of small sample size and lack of a control group, our study provides preliminary evidence supporting prospective studies to refine patient selection and long-term outcomes.

## Supplementary Information

Supplementary Material 1.

## Data Availability

All data generated or analysed are available from the corresponding author on reasonable request.

## Abbreviations

ED: Esophageal diverticulum
ZD: Zenker diverticulum
POEM: Peroral endoscopic myotomy

## References

[1] Yang J, Zeng X, Yuan X, et al. An international study on the use of peroral endoscopic myotomy (POEM) in the management of esophageal diverticula: the first multicenter D-POEM experience. Endoscopy. 2018;51(4):346–9. 30453378 10.1055/a-0759-1428

[2] Hiroki S, Manabu T, Kazuya T et al. Esophageal Diverticulum - Indications and Efficacy of Therapeutic Endoscopy. %J Internal medicine (Tokyo, Japan). 2022, 61(7): 943-9. 10.2169/internalmedicine.8196-21 PMC9038461 35370253

[3] Sato H, Fujiyoshi Y, Abe H, et al. Development of Dilated Esophagus, Sigmoid Esophagus, and Esophageal Diverticulum in Patients With Achalasia: Japan Achalasia Multicenter Study. J Neurogastroenterol Motil. 2022;28(2):222–30. 35362448 10.5056/jnm21188 PMC8978127

[4] Barbieri LA, Parise P. Treatment of Epiphrenic Diverticulum: How I Do It. J Laparoendosc Adv Surg Tech A. 2020;30(6):653–8. 32315575 10.1089/lap.2020.0165

[5] Dado G, Bresadola V, Terrosu G, et al. Diverticulum of the midthoracic esophagus: pathogenesis and surgical treatment. Surg Endosc. 2002;16(5):871. 11997849 10.1007/s00464-001-4217-7

[6] Law R, Katzka D A. Baron T H. Zenker’s Diverticulum. Clin Gastroenterol Hepatol, 2013;12(11):1773–1782. 10.1016/j.cgh.2013.09.016 24055983

[7] Shrigiriwar A, Mony S, Fayyaz F et al. Clinical outcomes of peroral endoscopic myotomy with and without septotomy for management of epiphrenic diverticula: an international multicenter experience (with video). Gastrointest Endosc, 2024;100(5):1012–1020. 10.1016/j.gie.2024.05.010 38795736

[8] Inoue H, Onimaru M. [Endoscopic Treatment for Esophageal Achalasia:Per-oral Endoscopic Myotomy(POEM)]. Kyobu Geka. 2024;77(10):896–901. 39617391

[9] Andolfi C, Wiesel O Fisichella PM. Surgical Treatment of Epiphrenic Diverticulum: Technique and Controversies. J Laparoendosc Adv Surg Tech A. 2016;26(11):905–10. 27631419 10.1089/lap.2016.0365

[10] Bremner C G Rivkinl, Bremner C H. Pathophysiology of mid-oesophageal and epiphrenic diverticula of the oesophagus. S Afr Med J. 1984;66(4):127–9. 6429868

[11] Herbella F A M, Patti MG. Modern pathophysiology and treatment of esophageal diverticula. Langenbecks Arch Surg. 2011;397(1):29–35. 21887578 10.1007/s00423-011-0843-2

[12] Ekberg O Nylanderg. Lateral diverticula from the pharyngo-esophageal junction area. Radiology. 1983;146(1):117–22. 6401363 10.1148/radiology.146.1.6401363

[13] Cook I J, Gabb M. Pharyngeal (Zenker’s) diverticulum is a disorder of upper esophageal sphincter opening. Gastroenterology. 1992;103(4):1229–35. 1397879 10.1016/0016-5085(92)91508-2

[14] Maydeo A, Patil G K Dalala. Operative technical tricks and 12-month outcomes of diverticular peroral endoscopic myotomy (D-POEM) in patients with symptomatic esophageal diverticula. Endoscopy. 2019;51(12):1136–40. 31614371 10.1055/a-1015-0214

[15] Wessels E M, Schuitenmaker J M, Bastiaansen B A J, et al. Efficacy and safety of peroral endoscopic myotomy for esophageal diverticula. Endosc Int Open. 2023;11(5):E546–52. 37251790 10.1055/a-2071-6744 PMC10219786

[16] Hernández Mondragón OV, Solórzano Pineda M O, Blancas Valencia J M. Zenker’s diverticulum: Submucosal tunneling endoscopic septum division (Z-POEM). Dig Endosc. 2017;30(1):124. 28875504 10.1111/den.12958

[17] Kamal F, Khan M A, Lee-Smith W, et al. Peroral Endoscopic Myotomy Is a Safe and Feasible Option in Management of Esophageal Diverticula: Systematic Review and Meta-Analysis. Dig Dis Sci. 2020;66(10):3242–9. 33123940 10.1007/s10620-020-06678-5

[18] Tapias L F, Morse C R, Mathisen D J, et al. Surgical Management of Esophageal Epiphrenic Diverticula: A Transthoracic Approach Over Four Decades. Ann Thorac Surg. 2017;104(4):1123–30. 28847539 10.1016/j.athoracsur.2017.06.017

[19] Albers D V Kondoa, Bernardo W M, et al. Endoscopic versus surgical approach in the treatment of Zenker’s diverticulum: systematic review and meta-analysis. Endosc Int Open. 2016;4(6):E678–86. 27556078 10.1055/s-0042-106203 PMC4993875

[20] Matthews B D, Nelms C D, Lohr C E et al. Minimally invasive management of epiphrenic esophageal diverticula. Am Surg, 2003;69(6):465–470. 12852502

[21] Nabi Z, Chavan R. Per-oral Endoscopic Myotomy with Division of Septum (D-POEM) in Epiphrenic Esophageal Diverticula: Outcomes at a Median Follow-Up of Two Years. Dysphagia. 2021;37(4):839–47. 34212259 10.1007/s00455-021-10339-8

[22] Steinway S, Zhang L, Amundson J, et al. Long-term outcomes of Zenker’s peroral endoscopic myotomy (Z-POEM) for treatment of Zenker’s diverticulum. Endosc Int Open. 2023;11(6):E607–12. 37397859 10.1055/a-2067-9105 PMC10310448

[23] Caso R, Chang H, Marshall MB. Evolving Options in Management of Minimally Invasive Diverticular Disease: A Single Surgeon’s Experience and Review of the Literature. J Laparoendosc Adv Surg Tech A. 2019;29(6):780–4. 30620240 10.1089/lap.2018.0711

[24] Lian J, Xu A, Chen T, et al. Kill two birds with one stone: one submucosal tunnel for achalasia combined with large epiphrenic diverticulum. Endoscopy. 2023;55(S 01):E1073–4. 37734413 10.1055/a-2163-1924 PMC10777683

