# Supplementary material for: POEM-based endoscopic myotomy for esophageal diverticulum: a case series study
Source: BMC Gastroenterol. 2026 May 12;26:417. doi: 10.1186/s12876-026-04910-6 (PMC13340009; doi:10.1186/s12876-026-04910-6)
Supplement: Supplementary file 1 — Supplementary Material 1. [file 12876_2026_4910_MOESM1_ESM.docx]

**Supplementary Material**

**Representative Cases**

**Case #1**

A 44-year-old woman presented with a 15-day history of progressive upper abdominal distension and dysphagia. Endoscopy revealed a large diverticulum located near the cardia with food retention, and barium esophagography confirmed a 3.1 × 2.2 cm epiphrenic diverticulum with a narrow 1.1 cm neck (Supplementary Figure S1). Coexisting achalasia was diagnosed based on a narrowed cardia and impaired esophageal emptying.

Under general anesthesia with endotracheal intubation, a combined POEM and D-POEM procedure was performed. A submucosal injection was placed 1 cm above the diverticulum, followed by creation of a mucosal entry using a running water knife. The submucosal tunnel was directed toward and along the diverticular ridge, with dissection centered on the distal/free margin of the septum to expose the septal musculature. Myotomy was performed from the diverticular apex to its basal region and was extended approximately 2–3 cm beyond the lower esophageal sphincter. Hemostasis was achieved using hot biopsy forceps, and the tunnel entry was closed with metal clips.

During tunneling, two submucosal white lesions (0.5 cm and 0.8 cm) were observed at 32 cm from the incisors (Supplementary Figure S2A). The lesions were completely resected within the tunnel and were later confirmed as leiomyomas on pathological and immunohistochemical examination.

The patient recovered uneventfully and was discharged on day 5. Follow-up endoscopy at 6 months demonstrated a fully flattened diverticulum without residual food or liquid, and the cardia showed smooth passage (Supplementary Figure S2 B–D). The patient remained asymptomatic.

**Case #4**

A 57-year-old woman was referred for reflux, choking cough, and esophageal obstruction feeling initially misinterpreted as gastroesophageal reflux disease. Endoscopy revealed a deep ZD located 17 cm from the incisors with significant food retention. During general anesthesia, a submucosal injection was made 14 cm from the incisors, and a mucosal incision created the tunnel entrance. The diverticular septum was gradually dissected, exposing the muscular ridge (Supplementary Figure S3C). A complete septostomy was then performed using a gold knife (Supplementary Figure S3D), and the incision entry was closed with metal clips.

The patient recovered without complications and was discharged on postoperative day 4. Follow-up endoscopy at 6 months showed a flattened esophageal inlet and absence of food retention (Supplementary Figure S3 A–B). The patient reported complete resolution of choking episodes and effortless eating.

**Case #7**

An 80-year-old woman presented with a 6-month history of dysphagia and prolonged mealtime, complicated by prior pneumonia. CT revealed mid-esophageal dilation, and endoscopy confirmed a 2.5 cm mid-ED located 25 cm from the incisors with food stasis (Supplementary Figure S4A).

Under general anesthesia, a submucosal injection was made 4 cm above the diverticulum using saline mixed with indigo carmine and epinephrine. A mucosal incision was created, followed by careful expansion of the submucosal tunnel toward the diverticular ridge, with dissection centered on the septal/muscular ridge. The tunnel enabled full visualization of the muscular ridge of the diverticulum (Supplementary Figure S4B), which was then incised completely (Supplementary Figure S4C). Hemostasis was achieved using electrocautery, and the mucosal entry was closed with clips (Supplementary Figure S4D).

The postoperative course was uneventful. At 6 months, endoscopy confirmed a completely collapsed mid-esophageal diverticulum, and the patient reported full resolution of dysphagia without recurrence.

**Supplementary Figure Legends**


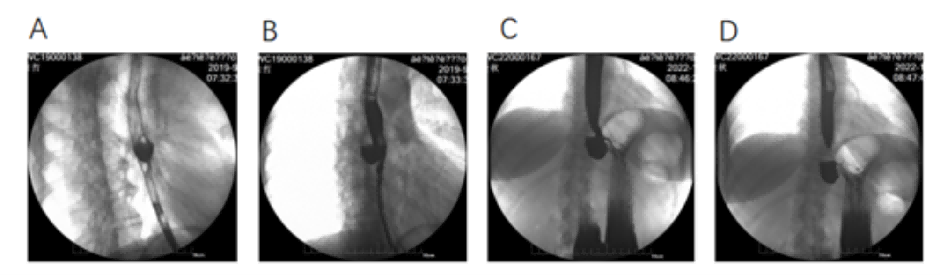


**Supplementary Figure S1.** Barium meal perspective of a lower esophageal diverticulum. A-B. Supraseptal diverticulum without achalasia. C-D. Supraseptal diverticulum with achalasia.


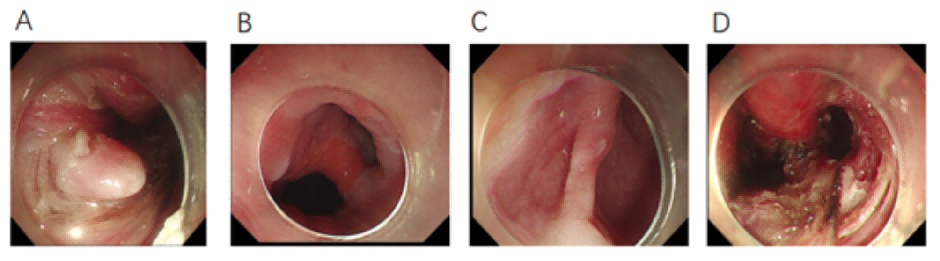


**Supplementary Figure S2.** Epiphrenic diverticulum in patient #1. A. Esophageal leiomyoma was found after the tunnel was established and resected. B. Supraseptal diverticulum C. A smaller diverticulum is seen next to a larger diverticulum D. The diverticular septum was completely divided.


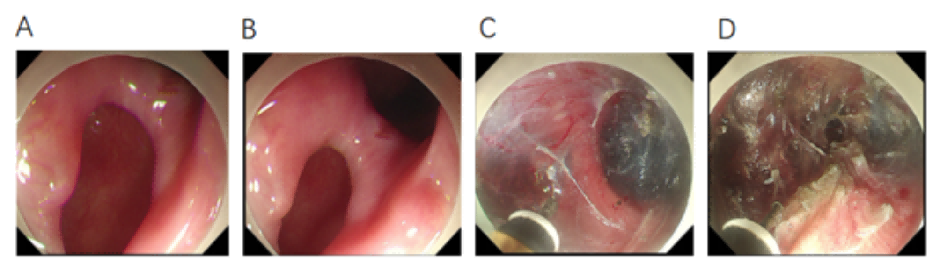


**Supplementary Figure S3.** Intraoperative endoscopic images of patient #4 with Zenker diverticulum. A–B. Zenker diverticulum. C. Exposure of the diverticular septum after creation of the submucosal tunnel. D. Septal myotomy of the diverticulum.
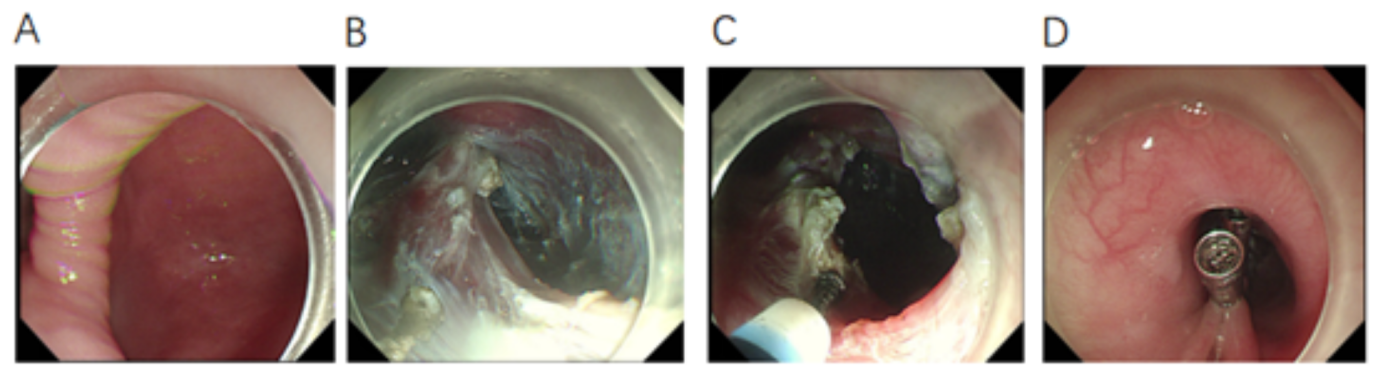


**Supplementary Figure S4.** Mid-esophageal diverticulum in patient #7. A. Mid-esophageal diverticulum. B. Exposure of the muscular ridge after tunnel creation. C. Myotomy of the diverticular muscular ridge. D. Closure of the mucosal entry with endoscopic clips.
